# Ethical challenges in the neonatal intensive care units: perceptions of physicians and nurses; an Iranian experience

**Published:** 2015-02-04

**Authors:** Maliheh Kadivar, Ziba Mosayebi, Fariba Asghari, Pari Zarrini

**Affiliations:** 1 Professor, Department of Pediatrics, Division of Neonatology, Children’s Medical Center, Tehran University of Medical Sciences, Tehran, Iran;; 2 Associate Professor, Department of Pediatrics, Division of Neonatology- Children’s Medical Center, Tehran University of Medical Sciences, Tehran, Iran;; 3 Associate Professor, Medical Ethics and History of Medicine Research Center, Tehran University of Medical Sciences, Tehran, Iran;; 4 Neonatologist, Department of Pediatrics, Division of Neonatology- Children’s Medical Center, Tehran University of Medical Sciences, Tehran, Iran.

**Keywords:** ethical issues, neonatal intensive care unit (NICU), nursing ethics, hospital ethics committee

## Abstract

The challenging nature of neonatal medicine today is intensified by modern advances in intensive care and treatment of sicker neonates. These developments have caused numerous ethical issues and conflicts in ethical decision-making. The present study surveyed the challenges and dilemmas from the viewpoint of the neonatal intensive care personnel in the teaching hospitals of Tehran University of Medical Sciences (TUMS) in the capital of Iran.

In this comparative cross-sectional study conducted between March 2013 and February 2014, the physicians’ and nurses’ perceptions of the ethical issues in neonatal intensive care units were compared. The physicians and nurses of the study hospitals were requested to complete a 36-item questionnaire after initial accommodations. The study samples consisted of 284 physicians (36%) and nurses (64%). Content validity and internal consistency calculations were used to examine the psychometric properties of the questionnaire. Data were analyzed by Pearson's correlation, t-test, ANOVA, and linear regression using SPSS v. 22.

Respecting patients’ rights and interactions with parents were perceived as the most challenging aspects of neonatal care. There were significant differences between sexes in the domains of the perceived challenges. According to the linear regression model, the perceived score would be reduced 0.33 per each year on the job.

The results of our study showed that the most challenging issues were related to patients’ rights, interactions with parents, communication and cooperation, and end of life considerations respectively. It can be concluded, therefore, that more attention should be paid to these issues in educational programs and ethics committees of hospitals.

## Introduction

Today, the whole health system is faced with issues such as the rapid advancement of technology, economic changes, resource constraints, and problems in health care delivery (1). Due to the nature of their career, health team members are constantly exposed to ethical issues and challenges such as abortion, end of life care, and medical errors (1, 2). There are fundamental differences between adults and children, and therefore the issues related to children and infant care and ethical decision-making are quite different.

Although neonatal wards are always filled by moral conflicts, amazing advances in the care of critically ill neonates have led to challenging established standards and clear procedures in neonatal care (3 , 4). These advances have caused more conflicts in ethical decision-making and developed more ethical challenges. In a study on 70 physicians and caregivers, 210 ethical problems were reported, which means an average of three problems per person. In other words, each caregiver was confronted by more than one ethical problem on a regular basis (5). 

It is clear that a single person or group of people cannot make the right decisions without proper training, and therefore hospital ethics committees and ethics consultation were created (3 , 6). The latter has been introduced as a mechanism approved by ethics experts to help in situations of moral conflict (6 , 7). Similarly, important decisions are made by ethics committees based on the long established practices in developed countries (8). However, ethics committees and ethics consultation have only been introduced to the Iranian health system in recent years. 

Although some studies have been conducted to identify the most common ethical issues of the medical system in Iran (9), less attention has been paid to issues in the field of neonatology. This survey evaluated these matters from the viewpoint of the neonatal intensive care personnel in the teaching hospitals of Tehran University of Medical Sciences (TUMS) in the capital of Iran. The role of the hospitals’ ethics committees and ethics consultation were also assessed in the present study.

## Materials and methods

This was a comparative cross-sectional study conducted between March 2013 and February 2014. The study population consisted of physicians (including neonatologists, fellows of neonatology, and residents of pediatrics) and nurses of the neonatal care units level II and III of the teaching hospitals affiliated with TUMS. The samples were collected from the neonatal units of seven teaching hospitals using convenience sampling method. 

The first phase of the study was to design a valid and reliable tool for assessing the ethical issues in neonatal units. First, related sources of data, including theses, dissertations and electronic databases (PubMed, Science Direct and Proquest) were examined for a suitable questionnaire. The used keywords were ethical, issues, dilemmas, challenges, neonatology, and NICU. The research team designed a new questionnaire for the purpose of this study. Common ethical issues and challenges were extracted through interviews with experts in the field of ethics and neonatology, and literature review, and the results were gathered together as a questionnaire. After six revisions of the questionnaire by the research team and consultations with ethics experts, the first draft was designed with 60 items. An expert panel consisting of two neonatal care professors, two medical ethics experts and three members of the research team revised the questionnaire further and omitted, modified or merged some questions. The final instrument included 36 questions related to ethical issues and consultation. In the pilot phase, a sample of 20 nurses and physicians completed the questionnaire and the final adjustment was performed accordingly. 

In the main phase of the study a three-part questionnaire was prepared as reported above. The first part contained demographic questions, including variables such as age, gender, and work experience. The main part consisted of the first 36 questions of the developed questionnaire, designed to assess the ethical issues and challenges in neonatal care. Ethical issues were divided into four domains: communication and cooperation, patients’ rights, end of Life considerations, and interaction with patients. The questions are presented in table 1. 

The next 10 questions examined the participants’ viewpoints regarding hospital ethics committees and ethics consultation. The questions of this part are presented in table 2. 

**Table 1:** The ethical issues in neonatal intensive care units

Domain	Item No.	Items
Communication and Cooperation	2	Variation of medical opinions on different shifts
	3	Declining of physicians’ decisions by nurses
	8	Absence of senior authorities in NICU
	17	Inappropriate behavior among medical staff
	18	Improper management of medical errors on neonates
	19	Unreported ethical issues
	24	Lack of coordination between NICU and other wards
Patients’ Rights	1	Patient confidentiality in NICU
	14	Interest in some patients leading to unfair services to others
	22	Neglecting to obtain informed consent
	23	Lack of respect for patient privacy (Such as taking pictures without consent)
	25	Lack of respect for the rights of infants during residency training
	26	Lack of respect for the rights of infants in medical research
Interaction With Parents	4	Improper parent requests that are contrary to medical advice
	5	Disagreement between parents in choosing the treatment
	6	Refusal of parents to give correct information about the infants’ conditions
	7	Parents’ failure to understand the infants’ conditions
	16	Discharge Against Medical Advice (DAMA) due to financial problems
	20	Differences in language, culture and religion
	21	Lack of parental competency in decision making
End of Life Considerations	9	Lack of decision-making protocols (Such as Do Not Resuscitate Order, Discharge Against Medical Advice, etc.)
	10	Futile care
	11	Treatment of neonates with congenital abnormalities or poor prognoses
	12	Decisions based on the type and severity of illness, age and birth weight
	13	Devoting the resources to children with poor prognoses
	15	Using infants’ prognoses as a basis for NICU admission

*NICU= neonatal intensive care unit.*

**Table 2:** The main strategies in confronting ethical issues

Item No.	Items
27	Formulating decision-making protocols (Such as Do Not Resuscitate Order)
28	Providing the necessary information and proper communication
29	Caregivers’ accountability
30	Helpfulness of hospital ethics committees
31	Functions of hospital ethics committees
32	Use of hospital ethics committees
33	Helpfulness of hospital ethics committees’ comments
34	Helpfulness of hospital ethics committees on the occasion of conflicts between patients’ families and hospital staff
35	Helpfulness of hospital ethics committees in reducing ethical issues in NICU
36	Attending related courses

*NICU= neonatal intensive care unit.*

Each question had a 5-point Likert assessment. To achieve a score of 100, the sum of each domain’s question scores were divided by the maximum attainable score in that domain and then the result was multiplied by 100. The content validity of the questionnaire was assessed by an expert panel of twelve bioethicists and neonatologists. The Cronbach's alpha coefficient was calculated for internal consistency (α = 0.89 and α = 0.77 respectively).

Inclusion criteria were at least three months’ experience of working in neonatal intensive care units (NICUs) and agreement to complete the questionnaire. The main researcher visited all selected NICUs and asked physicians and nurses to complete the questionnaire in person. After a full explanation of the study purpose, the participants were asked to complete the questionnaire carefully. Participation in the study was voluntary and a written consent was obtained after the initial briefing. 

Two hundred and eighty six nurses and physicians out of a total of 350 completed the questionnaire. The response rate was 81.71 percent. The questionnaires were completed anonymously, and all personal data were removed. The researcher assured all participants that the data was confidential and would only be used for research purposes. Data were entered into SPSS software v. 22. Means and standard deviations of each question score and domain were reported. Data were analyzed by Pearson's correlation, t-test, ANOVA, and linear regression. 

## Results

The results showed that the majority of subjects were female, married and Muslim. The majority of nurses had a bachelor's degree and most physicians were residents of pediatrics. Most of the participants had formerly participated in an ethics education program, and the most common educational method had been the workshop. The demographic characteristics of the study subjects are presented in table 3. 

**Table 3:** Demographic characteristics of study participants

Variable		Value number (Percentage) or Mean ± SD
Sex	Female	259 (91.2%)
	Male	25 (8.8%)
Age		34.14±6.57
Marital Status	Married	187 (66.1%)
	Single	96 (33.9%)
Religion	Muslim	280 (98.9%)
	Other	3 (1.1%)
Job	Physician	102 (36%)
	Nurse	183 (64%)
Education	Bachelor	160 (55.9%)
	Master of Science	23 (8%)
	Resident	51 (17.8%)
	Pediatrician	29 (10.1%)
	Neonatologist	10 (3.5%)
	Faculty Member	13 (4.5%)
Work Experience (Month)		60.66±66.
Previous ethics education	Yes	147 (51.4%)
	No	139 (48.6%)
Workshop time (Hours)		15.01±18.34

**Figure 1:**
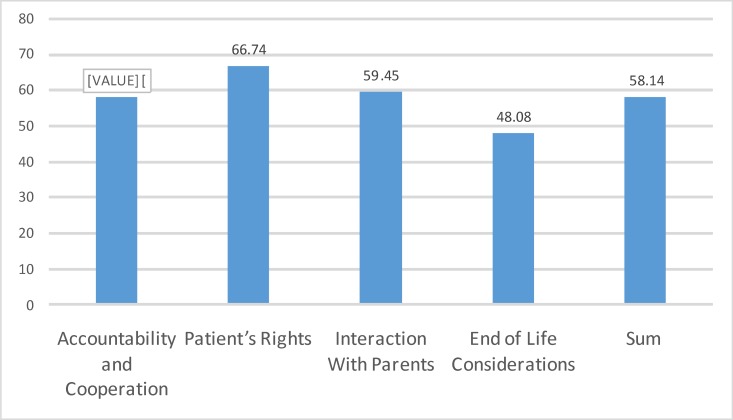
Perceived Issues by Physicians and Nurses

According to the caregivers in this study, the most important problems were in the ​​patients’ rights domain. After that, lack of appropriate interaction with parents and sense of duty, and cooperation had similar importance. End of life considerations were the least perceived challenges. The caregivers also believed that one major problem was interest in a specific patient and unfair services to other patients (item No. 14). Disagreement between parents in choosing the treatment (item No. 5) was perceived as the next most challenging issue. Confidentiality (item No. 1) and problems with patient privacy (item No. 23) were the next most important issues. Doctors faced a significantly higher number of challenges than nurses *(P < *0.05(. The ethical issues from the perspective of the caregivers and the comparison between nurses and physicians are shown in table 4 and figure 1. 

**Table 4:** The ethical issues in neonatal intensive care units and the comparison between physicians and nurses

Domain	Item No.	All		Physicians		Nurses		P Value
		Mean	SD	Mean	SD	Mean	SD	
Communication and Cooperation	2	2.80	1.06	2.96	1.06	2.70	1.05	0.05
	3	3.21	1.15	3.11	1.21	3.26	1.12	0.27
	8	3.29	1.11	3.54	1.09	3.15	1.10	0.004
	17	3.01	1.19	3.17	1.22	2.92	1.18	0.08
	18	2.81	1.12	2.92	1.17	2.74	1.10	0.19
	19	2.83	1.06	2.99	1.12	2.74	1.02	0.06
	24	2.39	1.12	2.49	1.20	2.33	1.07	0.27
	Sum	58.10	14.8	60.53	15.25	56.74	14.55	0.03
Patients’ Rights	1	3.58	1.06	3.72	1.07	3.51	1.05	0.10
	14	3.73	1.13	3.78	1.09	3.71	1.16	0.63
	22	2.88	1.12	3.17	1.15	2.72	1.07	0.001
	23	3.48	1.20	3.81	1.14	3.30	1.21	0.001
	25	3.05	1.14	3.42	1.12	2.85	1.11	0.00
	26	3.29	1.12	3.65	1.10	3.09	1.09	0.00
	Sum	66.74	14.93	71.81	14.08	63.90	14.68	0.00
Interaction With Parents	4	3.15	1.10	3.28	1.07	3.08	1.12	0.13
	5	3.64	1.09	3.81	1.09	3.54	1.09	0.05
	6	2.78	1.14	2.81	1.21	2.77	1.11	0.77
	7	2.16	0.85	2.15	.87	2.17	.85	0.82
	16	3.19	1.11	3.28	1.07	3.13	1.13	0.27
	20	3.16	1.20	3.37	1.26	3.04	1.17	0.02
	21	2.73	1.14	2.88	1.20	2.65	1.11	0.09
	Sum	59.45	14.36	61.64	14.33	58.22	14.27	0.05
End of Life Considerations	9	2.50	1.02	2.50	1.00	2.50	1.03	0.95
	10	2.66	1.14	3.02	1.12	2.45	1.10	0.00
	11	2.27	1.01	2.28	0.98	2.27	1.02	0.91
	12	2.10	1.10	2.26	1.16	2.01	1.06	0.05
	13	2.02	1.00	2.10	1.05	1.98	0.96	0.35
	15	2.87	1.18	3.14	1.20	2.73	1.15	0.005
	Sum	48.08	12.70	51.00	11.89	46.45	12.87	0.003
Sum		58.14	11.74	61.23	11.34	56.41	11.64	0.001

*P *> 0.05was considered significant.

Concerning the need for ethics consultation, questions related to ethics committees were allocated the highest priority. In table 5, the most important strategies and a comparison between doctors and nurses are presented. There were significant differences between men and women in perceived issues *(P < *0.05). 

**Table 5:** The main strategies in confronting ethical issues and the comparison between physicians and  nurses

Item No.	All		Physicians		Nurses		P Value
	Mean	SD	Mean	SD	Mean	SD	
27	2.14	1.05	2.26	1.08	2.08	1.03	0.15
28	1.88	0.79	1.91	0.81	1.86	0.78	0.61
29	1.87	0.92	1.90	0.99	1.86	0.89	0.69
30	2.02	1.01	2.22	1.21	1.90	0.85	0.00
31	3.48	1.04	3.47	1.08	3.49	1.01	0.87
32	3.96	1.02	3.96	0.99	3.96	1.04	0.99
33	3.56	1.05	3.49	0.98	3.60	1.08	0.37
34	2.29	1.14	2.30	1.10	2.29	1.17	0.93
35	2.48	1.14	2.51	1.20	2.46	1.12	0.72
36	2.33	1.06	2.58	1.15	2.19	0.99	0.004

*(Significant if P > 0.05)*

Men reported more challenges in the domains of patients’ rights and interaction with parents. There was a negative relationship between work experience and perceived issues *(P = *0.002, r = - 0.186). The relationships between age (*P *= 0.32, r = - 0.06), ethics education course duration (*P *= 0.75, r = - 0.03) and perceived issues were not significant. Marital status and educational level did not have a significant effect on perceived issues among physicians (*P *> 0.05). Moreover, nurses with a bachelor’s degree had a higher perception of ethical issues (*P *< 0.05). The relationships between ethical issues and demographic characteristics are presented in table 6.

**Table 6:** The relationship between demographic characteristics and perception of ethical issues

		Communication and Cooperation		Patients’ Rights		Interaction With Parents		End of Life Considerations		Ethical Issues Perception		
		Mean	SD	Mean	SD	Mean	SD	Mean	SD	Mean		SD
Gender	Female	57.55	14.62	66.11	14.72	58.49	14.06	47.92	12.86	57.56		11.57
	Male	62.28	16.72	72.80	16.40	67.77	14.48	49.73	11.46	63.29		12.43
	P Value	t=-1.52, df=282, p=0.12		t=-2.14, df=282, p=0.03		t=-3.14, df=282, p=0.002		t=-0.67, df=282, p=0.50		t=-2.34, df=282, p=0.01		
Education	Bachelor	57.85	14.25	65.62	13.81	58.91	14.33	47.12	12.63	57.46	11.25	
	Master of Science	48.81	14.43	51.88	15.16	53.41	13.10	41.73	13.81	49.13	11.88	
	P Value	t=2.84, df=181, p=0.005		t=4.40, df=181, p=0.00		t=1.73, df=181, p=0.08		t=1.88, df=181, p=0.06		t=3.29, df=181, p=0.001		
	Resident	62.96	14.84	74.24	14.39	60.00	14.64	52.61	10.52	62.38	11.26	
	Pediatrician	61.08	16.24	67.81	12.79	64.82	15.08	48.73	11.31	60.79	10.90	
	Neonatologist	54.57	14.90	72.00	15.88	57.14	10.94	46.33	12.21	57.38	12.38	
	Professor	54.28	13.35	71.02	13.63	64.39	13.02	53.33	16.77	60.65	12.35	
	P Value	f=1.71, p=0.16		f=1.31, p=0.27		f=1.19, p=0.31		f=0.57, p=0.63		f=2.08, p=0.10		
Previous ethics education	Yes	56.90	13.87	65.71	14.28	57.95	13.48	47.89	12.67	57.14	11.10	
	No	59.36	15.84	67.84	15.56	61.02	15.11	48.29	12.76	59.21	12.33	
	P Value	t=1.39, df=181, p=0.16		t=1.20, df=181, p=0.22		t=1.81, df=181, p=0.07		t=0.27, df=181, p=0.78		t=1. 49, df=181, p=0.13		

*(Significant if P > 0.05)*

## Discussion

The main objective of the current study was to assess the most perceived ethical issues in neonatal intensive care units. Another objective was to determine the caregivers’ opinions about ethics consultation and hospital ethics committees. Finally, the perceptions of doctors and nurses were compared. According to the results, issues related to confidentiality and patient privacy were considered the most important. Although the pattern of perceived challenges was the same in both groups, physicians reported more ethical challenges than nurses, which may indicate their higher ethical sensitivity. Two other studies also indicated differences between these two groups, and a higher sensitivity to ethical issues in physicians (10,11). 

The first study was conducted in eight Western European countries: France, Germany, Great Britain, Italy, Luxembourg, the Netherlands, Spain and Sweden. One hundred and twenty-two neonatal intensive care units (NICUs) were recruited by census. A total of 1235 completed questionnaires from doctors and 3115 from nurses were returned. Similar to our study, both doctors and nurses participated, but only the findings related to doctors were reported. The results showed that most physicians reported having been involved at least once in setting limits to intensive care because of a baby’s incurable condition and/or poor neurological prognosis (10). 

In the second study all internal medicine, oncology and intensive care specialists and associated nurses (n = 532) employed at a university hospital in Croatia participated. The most frequent ethical dilemmas involved uncertain or impaired decision-making capacity, limitation of treatment at the end of life and disagreements among family members. Findings of this study showed that the two groups had similar opinions in their strategies (11).

Our results revealed some differences between women and men in their perception of ethical issues. The most challenging area for both genders was patients’ rights**. **In this regard, we found another study on 500 health professionals employed at the Mexican Institute of Social Security in Mexico City conducted in the form of structured interviews. The results showed that women attributed more importance to mutual respect, patient privacy and confidentiality, while men were more ethical in terms of providing up-to-date information and telling the truth (1). These findings indicate that women and men perceive different forms of ethical challenges. Nevertheless, conflicting results have been reported. There was a study on physicians and nurses engaged in geriatric and surgical care at a major hospital in Sweden that was conducted to compare differences in moral reasoning between men and women. The results showed no difference between men and women in this respect (12). Similarly, research conducted on Kuwaiti students did not indicate differences in patterns of moral reasoning between the genders (13). Another study on one hundred healthy volunteers recruited from students at the University of Milan showed that men tended towards utilitarian views in their moral judgments (14). Considering all the conflicting findings mentioned above, it seems that more research is needed in this area. 

In the present study, participants' attention to ethical issues decreased with increasing age and more experience. In a study of nurses’ perceptions, there was no significant relationship between age and level of participation and satisfaction with ethical decision making (15). We were also able to find two studies that showed no correlation between increasing age and ethical sensitivity. The first study was conducted on 156 accounting undergraduates employed in China, and the results showed no relationship between age and ethical sensitivity (16). The second was a study by Hassanpoor et al. in Kerman, and did not indicate a significant relationship between age and ethical sensitivity (17).

In a study conducted in Turkey, physicians who had more work experience showed more sensitivity to ethical issues (18). Our results showed that the perceived issues decreased with increasing age. This may be due to non-ethical work atmosphere. It seems that the lack of promotion of moral knowledge and compliance with hospital routines reduces ethical sensitivity. Nevertheless, further investigation seems to be necessary to confirm these results.

The field study confirmed that although the majority of participants had positive views about ethics committees, they did not have specific information about the dimensions of work, authority, and the role of these committees. Schneiderman et al. also showed that the majority of doctors, nurses and patients/guardians (87%) believed that ethics consultation can help resolve ethical conflicts. They concluded that ethics consultation may be helpful in settling conflicts, and is a useful technique to prevent unwanted treatments (19). Despite these advantages, studies have shown that clinical staff are often unaware of ethics committees or do not know how to seek their services (3,8,20). It is recommended that neonatal specialists, staff, and parents be aware of the potential role of ethics committees and use their services in cases of moral conflicts (3,21). 

Our results showed no relationship between previous ethics education and perceived issues. While participation in training programs may foster attention to ethical issues, results of studies have not confirmed this. Studies by Ersoy in Turkey showed that people who had received prior training in ethical issues had more knowledge and ethical sensitivity (18,22). A study in Taiwan showed, however, that training alone does not raise awareness of ethical issues among nurses, and ethical sensitivity should be internalized (23). We recommend that the effectiveness of current ethics education be further examined.

## Conclusion

Based on the current study results, the most important issues in the field of ethics were related to patients’ rights, interaction with parents, communication and cooperation, and end of life considerations respectively. Special attention should be paid to this aspect of training programs for residents and nurses. On the other hand, the effectiveness of current ethics training programs needs to be examined. The results also showed that caregivers are not adequately familiar with the concept of ethics consultation and the functions of hospital ethics committees. Meetings between different departments, courses on the role of hospital ethics consultation and ethics committees can be helpful in this area. Today, complex health systems need staff who pay attention to the ethical aspects of their work, are familiar with ethical principles, and can make proper decisions. Failure at any stage can cause non-ethical action. Based on the results of the present study, employees need to be more sensitized to the ethical aspects of their work. In conclusion, the decreased attention to ethics resulting from the increase in age and job experience is an important issue that needs to be further investigated. 
